# Exercise Training and Work Task Induced Metabolic and Stress-Related mRNA and Protein Responses in Myalgic Muscles

**DOI:** 10.1155/2013/984523

**Published:** 2012-12-05

**Authors:** Gisela Sjøgaard, Mette K. Zebis, Kristian Kiilerich, Bengt Saltin, Henriette Pilegaard

**Affiliations:** ^1^Institute of Sports Science and Clinical Biomechanics, University of Southern Denmark, 5320 Odense M, Denmark; ^2^The Copenhagen Muscle Research Centre and Centre of Inflammation and Metabolism, Department of Biology, University of Copenhagen, August Krogh Building, 2100 Copenhagen Ø, Denmark; ^3^Copenhagen Muscle Research Centre, Rigshospitalet, University of Copenhagen, 2100 Copenhagen Ø, Denmark

## Abstract

The aim was to assess mRNA and/or protein levels of heat shock proteins, cytokines, growth regulating, and metabolic proteins in myalgic muscle at rest and in response to work tasks and prolonged exercise training. A randomized controlled trial included 28 females with trapezius myalgia and 16 healthy controls. Those with myalgia performed ~7 hrs repetitive stressful work and were subsequently randomized to 10 weeks of specific strength training, general fitness training, or reference intervention. Muscles biopsies were taken from the trapezius muscle at baseline, after work and after 10 weeks intervention. The main findings are that the capacity of carbohydrate oxidation was reduced in myalgic compared with healthy muscle. Repetitive stressful work increased mRNA content for heat shock proteins and decreased levels of key regulators for growth and oxidative metabolism. In contrast, prolonged general fitness as well as specific strength training decreased mRNA content of heat shock protein while the capacity of carbohydrate oxidation was increased only after specific strength training.

## 1. Introduction 

It is well established that musculoskeletal disorders comprise one of the most common and costly public health problems in North America and Europe [1]. Especially, the prevalence of myalgia localized to the neck and shoulders in women is a growing problem both in the general population and in the industrial world, and trapezius myalgia is a major concern in jobs involving repetitive and monotonous work tasks [2]. The pathogenesis of work-related trapezius myalgia is not well understood. Several studies have revealed pathological mechanisms implicated in localized female trapezius myalgia. Special strain on type I fibers in females with trapezius myalgia compared with healthy controls [3], increased occurrence of ragged red fibers in trapezius muscle [4], disorganized mitochondrial pattern [3, 5], and a higher proportion of COX-negative fibers in patients with trapezius myalgia [5] have been reported, indicating that trapezius myalgia involves an impaired intramuscular environment. It is well known that the mRNA content for several metabolic and stress-related genes increases in human skeletal muscle in response to acute exercise [6–8]. However, repetitive work tasks may have a contrasting effect to exercise training on the mRNA content of those genes, and it has been postulated: “that sustained exposure to ergonomic stressors could disrupt the heat shock response and lead to sustained pathogenic levels of chaperone production, which in turn ultimately results in focal cell death” [9]. This still remains to be elucidated.

 Physical exercise training has been suggested as a treatment of musculoskeletal disorders [10–12], and regular training augments basal skeletal muscle protein and mRNA levels of important metabolic enzymes [8, 13]. However, the response to long-term exercise training on mRNA and protein content in females with work-related trapezius myalgia is unknown but can serve to tailor exercise treatments for this population group. Heat shock or stress proteins (HSPs) are considered to play an essential role in protecting cells from stress and preparing them to survive new environmental challenges. Thus, a number of studies have shown that HSP confers protection against cellular stresses including hyperthermia, hypoxia, ischemia, and reperfusion, which would otherwise lead to cell death [14–16]. As a molecular chaperone, HSP plays an important role in facilitating protein synthesis, folding, and assembly as well as in environmental adaptation and organism development [17]. In mammalian cells, the most highly induced proteins of the cellular stress response are the components of the protein family of 70 kDa, a group of closely related proteins that includes HSP72 and HSc70 [18]. While HSc70 is constitutively expressed, HSP72 is present in low quantities in unstressed cells and is thought to be principally stress-inducible [19–21]. A number of previous studies have shown that in response to stress, muscle HSP mRNA is rapidly upregulated [6, 7, 18, 22, 23]. However, the role of heat shock protein in trapezius myalgia remains to be investigated in response to strength training as well as endurance type of training, both of which have proven to reduce muscle pain [24]. The aim of the present study was to determine the mRNA content of heat shock proteins together with that of cytokines, growth regulating proteins, as well as mRNA and protein content of key enzymes in metabolism. 

We hypothesized that the trapezius muscle displays

1. different profiles regarding mRNA content of heat shock proteins, cytokines, growth regulating proteins, and metabolic proteins in females with trapezius myalgia (MYA) compared with healthy matched controls (CON);
2. impaired mRNA content of heat shock proteins, cytokines, growth regulating proteins, and oxidative metabolic proteins after acute repetitive work in MYA;
3. improved mRNA content of heat shock proteins and cytokines after 10 wks of specific strength training as well as general fitness training in MYA. Strength training will additionally affect the mRNA and protein content of growth regulating and metabolic proteins.

## 2. Subjects and Methods

### 2.1. Study Design

The present study was part of a case-control and randomized controlled trial previously described [25, 26]. A subgroup of 28 females out of the original 48 subjects with trapezius myalgia (MYA) participated in the present study together with 16 healthy females who served as matched controls at baseline, (CON). In the participants, the mean ± SD for age, height, and weight was 44 ± 9.8 years, 1.65 ± 0.061 m, 72 ± 15.0 kg for the MYA group and 44 ± 9.1 years, 1.68 ± 0.055 m, 70 ± 10.6 kg for the CON group, respectively, with no significant differences between groups. All subjects were employed in jobs with monotonous and repetitive work tasks, for example, assembly line work and office work. MYAs were clinically diagnosed with trapezius myalgia when fulfilling the following criteria: (1) trouble (pain or discomfort) for more than 30 days during the last year in the neck/shoulder region, (2) more than 30 days of trouble in maximally three body regions out of eight major body regions (neck/shoulder, low back, and left or right arm/hand, hip, and knee/foot) in order to exclude generalised musculoskeletal diseases, (3) the trouble should be at least “quite a lot” on an ordinal 5-step scale ranging from “a little” to “very much,” (4) the trouble should be frequent (at least once a week), and (5) the intensity of the trouble should be at least 2 on a scale from 0 to 9, where 0 is no pain and 9 is the worst imaginable pain. Additionally, a positive clinical diagnosis of trapezius myalgia included palpable tenderness and tightness in the trapezius muscle [25]. Tenderness was assessed on a scale from 0 to 2 at 7 sites in the trapezius area on left and right sides resulting in a maximum tenderness score of 28. Correspondingly, CON fulfilled the following criteria: (1) pain or discomfort for less than 8 days during the last year in the neck/shoulder region and (2) no more than three body regions with more than 30 days of trouble, and negative replies were requested regarding question (3) to (5) and no tenderness or tightness of the trapezius muscle. Exclusion criteria for all were serious conditions such as previous trauma or injuries, life threatening diseases, cardiovascular diseases, or arthritis in the neck and shoulder.

At baseline, all participants (CON and MYA) had biopsies taken from the trapezius muscle in the morning. On this day, 8 out of the 28 MYA participated in a standardized repetitive and stressful work protocol and had a second biopsy taken in the afternoon the same day. Finally, the 28 MYAs were randomized into 3 different intervention groups: specific strength training (SST), general fitness training performed as leg bicycling (GFT), and health counseling as a reference group (REF). The randomization was a balanced design accounting for age, body mass index, and neck/shoulder trouble. The intervention lasted 10 wks and the participants had a third biopsy taken at the end of the intervention.

All subjects were informed about the purpose and the content of the project and gave written informed consent to participate in the study, which conformed to the Declaration of Helsinki and was approved by the local ethical committee (KF 01-138/04). The study qualified for registration in the International Standard Randomized Controlled Trial Number register (ISRCTN 87055459).

### 2.2. Repetitive and Stressful Work Day

The participants reported to the laboratory in the morning and the first biopsy was taken. The participants rested in a sitting position for 2 hrs. The resting period was followed by a 40-minute repetitive, low-force exercise period performed unilaterally on a pegboard using the side where the biopsy was taken in the trapezius muscle. The pegboard task was designed as repetitive hand movement task where short metal sticks with a wooden handle (load of 100 g) were moved back and forth between standardized positions, each 30 cm apart on a pegboard (3 close and 3 distant positions). Thus, the sticks were repositioning six times during cycles of 12 hand movements at a frequency of 1 Hz (for more details see [25]). Following this exercise, the participants filled in a questionnaire on the computer, watched a movie, or did some reading for the following 2 hrs. Finally, a stressful task was performed in terms of a color-word-conflict test for 10 minutes using a mouse-operated computer by the same hand as the pegboard task (for details see [25]). A subsequent ~1 hr recovery period was allowed before the last biopsy was taken. The pre and post biopsy was thus separated by ~7 hrs. The participants were fed with standardized light meals at frequent set time points throughout the experiment to maintain blood glucose and digestion as constant as possible. During the resting periods, light reference test contractions were performed with a number of physiological measuring devices mounted, for example, EMG, NIRS, and blood pressure in order to evaluate muscle loading and physiological responses during the work tasks. These experimental conditions are *per se* stressful and imply light repetitive work. 

### 2.3. Interventions

In the SST and the GFT groups, supervised training was performed at a high intensity for 20 minutes 3 times per week for 10 wks. The REF group was allocated 1 hr per week for lectures giving information on activities promoting general health (for details see [26]). 

SST performed supervised high-intensity-specific strength training locally for the neck and shoulder muscles with five different dumbbell exercises (1-arm row, shoulder abduction, shoulder elevation, reverse flyes, and upright row). Relative loadings were progressively increased from 12 repetitions maximum (RM) (~70% of maximal intensity) at the beginning of the training period towards 8 RM (~80% of maximal intensity) during the later phase. Three of the five different exercises with three sets per exercise were performed during each training session in an alternating manner, with shoulder-elevation being the only exercise that was performed during each session. 

GFT performed high-intensity general fitness training on a Monark bicycle ergometer for 20 min at relative workloads of 50–70% VO_2_-max. The subjects bicycled in an upright position without holding onto the handlebars. It was emphasized that the subjects in GFT should relax the shoulders during training. A relative workload of 50% of VO_2_-max was used during the initial training sessions and the intensity was progressively increased towards 70% during the following weeks and maintained at that intensity throughout the remaining training period. Heart rate was recorded in each subject by a heart rate monitor (Polar Sport Tester, Kempele, Finland) during each training session to adjust the workload to meet the intended relative level. 

REF received an equal amount of attention as the physical training intervention groups but not offered any physical training. These females received health counselling on group level and individual level with regard to work place ergonomics, diet, health, relaxation, and stress management for a total of up to 1 hr per week. Some lectures lasted up to 1 hr and could thus not be performed regularly three times a week to keep the total allocated time equal between intervention groups.

### 2.4. Biopsies

Muscle biopsy samples were taken from the upper part of the descending portion of the trapezius muscle. Baseline muscle biopsies were taken (~2.5 hrs after breakfast) using the percutaneous needle biopsy technique with suction after careful inspection ultrasound images taken the same morning as described previously [27]. Samples were immediately frozen in liquid nitrogen and transferred to −80°C for storage. In the afternoon the same day following repetitive and stressful tasks performed by the MYA group the second biopsy was taken approximately 1 hr after the last work task. The two biopsies were taken through the same skin incision at an angle such that the two sampling sites were separated as much as possible. Finally, the third biopsy was taken in the morning corresponding to the first biopsy and within 48–72 hrs after completion of the last training session of the 10 wks interventions of the MYA.

In total, 77 biopsies were taken (41 at baseline, 8 after the standardized work day, and 28 after the intervention study). Due to the fact that the size of the biopsies taken in some cases was too small to undergo all analysis presented in this study, the number of participants will be given in parentheses for each measurement. 

### 2.5. Outcome Measures

In all, 14 genes were analyzed in MYA and CON at baseline, and for only MYA also after an 7 hrs workday as well as following 10 wks training (see Table 1 in alphabetic order) for (1) heat shock proteins: HSP72, HSc70, and HSP3, where the latter is a member of the heme oxygenase family also termed HO-1, (2) cytokines: IL-6 (interleukin-6) and TNF-*α*  (tumor necrosis factor alpha), (3) growth related proteins: IGF-1Ea (insulin-like growth factor-1) and myostatin, and (4) metabolism/metabolic regulation: PGC-1*α*  (peroxisome proliferator-activated receptor gamma coactivator 1*α*), and HIF-1*α*  (hypoxia-inducible factor-1), GLUT4, GS (glycogen synthase), LDH-A, and LDH-B (lactate-dehydrogenase A and B).

Additionally, 5 key metabolic proteins were analyzed at baseline for MYA and CON, and for MYA only following 10 wks of training: cyt c (cytochrome c), PDH-E1*α*  (pyruvate dehydrogenase E1*α*), GLUT 4, GS, and HKII (hexokinase II).

#### 2.5.1. Muscle Lysate

Muscle pieces (~15 mg) were homogenized in an ice-cold buffer (10% glycerol, 20 mM Na-pyrophosphate, 150 mM NaCl, 50 mM HEPES, 1% NP-40, 20 mM *μ*-glycerophosphate, 10 mM NaF, 1 mM EDTA, 1 mM EGTA, 2 mM PMSF, 10 *μ*g/mL aprotinin, 10 *μ*g/mL leupeptin, 2 mM Na_3_VO_4_, 3 mM benzamidine, pH 7.5) for 20 s using a homogenizer (PT 3100, Kinematica, Littau-Lucerne, Switzerland). Homogenates were rotated end-over-end for 1 h at 4°C. Lysates were generated by centrifugation (17,500 g) for 20 min at 4°C. Protein content in lysates was measured by the bicinchoninic acid method (Pierce Chem Comp, IL, USA). 

#### 2.5.2. SDS-PAGE and Western Blotting

Protein expression was measured in muscle lysate by SDS-PAGE (Tris *·* HCl 7.5–15% gel, Bio-Rad, Denmark) and western blotting using polyvinylidene difluoride (PVDF) membrane and semidry transfer. After the transfer, the PVDF membrane was blocked over night at 4°C. The membrane was incubated with primary antibody for 2 hrs at room temperature followed by incubation with peroxidase-conjugated secondary antibody 1 h at room temperature. Immobilon Western (Millipore, Bedford, MA, USA) was used as a detection system. Bands were visualized using an Eastman Kodak Image Station 2000MM. Bands were quantified using Kodak Molecular Imaging Software v.4.0.3, and specific protein content is expressed in units relative to control samples loaded on each gel. Protein level of the PDH-E1*α*  subunit [28] and GS [29] was determined as previously described. Cyt C protein content was determined by blocking in TBS with Tween (TBST) + 3% BSA and primary antibody from BD Biosciences (Pharmingen, San Diego, CA, USA). HKII protein content was determined by blocking in TBST + 3% skim milk and primary antibody from Alpha Diagnostic international (San Antonio, TX, USA). GLUT4 protein content was determined by blocking in TBST + 2% skim milk and primary antibody from Chemicon (AB1346). Secondary antibodies used were all species-specific horseradish peroxidase-conjugated immunoglobulins (DakoCytomation, Glostrup, Denmark).

#### 2.5.3. RNA Isolation and Reverse Transcription

Total RNA was isolated from 5–15 mg of muscle tissue as previously described [6] with RNA resuspended (1 *μ*L per mg original tissue) in diethyl pyrocarbonate-treated H_2_O containing 0.1 mM EDTA. Reverse transcription (RT) was performed using the Superscript II RNase H^−^ system with Oligo dT (Invitrogen, Carlsbad, CA, USA) as previously described [6]. RT products originating from 1.5 *μ*g total RNA were diluted in nuclease-free H_2_O to a total volume of 95 *μ*L.

#### 2.5.4. PCR

The mRNA content of selected genes was determined by fluorescence-based real-time PCR (ABI PRISM 7900 Sequence Detection System, Applied Biosystems, Carlsbad, CA, USA). Forward (FP) and reverse (RP) primers and TaqMan probes were designed from human-specific sequence data (Entrez-NIH and Ensembl, Sanger Institute) using computer software (Primer Express, Applied Biosystems). The primers and probe sequences used are given in Table 1. The probes were 5′ 6-carboxyfluorescein (FAM) and 3′ 6-carboxy-N,N,N′,N′-tetramethylrhodamine (TAMRA) labelled. Prior optimization was conducted determining optimal primer concentrations, probe concentration, and verifying the efficiency of the amplification. PCR amplification was performed in triplicates in a total reaction volume of 10 *μ*L as previously described [30].

#### 2.5.5. Determination of Single-Stranded cDNA Content

The amount of single-stranded DNA (ssDNA) was determined in the RT samples using the OliGreen reagent (Molecular Probes, The Netherlands) as previously described [30]. In brief, samples were analyzed in a 96-well white microplate (Thermo Labsystems, Finland) with a total reaction volume of 200 *μ*L in each well. Each sample was run in triplicate with 5 *μ*L RNAse-treated cDNA sample, 95 *μ*L TE and 100 *μ*L of OliGreen reagent in each well. For further analytical details please see [30].

### 2.6. Statistical Analysis

All values are represented as median (range). Intergroup comparisons were tested by the Mann-Whitney  *U*  test. Evaluations of intra-group differences in protein content between two related samples were evaluated by the Wilcoxon-signed rank test for paired samples. Because no significant difference existed in mRNA content of selected genes at baseline between the three randomized MYA groups (SST, GFT, and REF), all data were pooled and compared with the respective groups after the intervention by use of the Mann-Whitney  *U*  test. All statistical analyses were performed using SPSS 12.0 for Windows (SPSS, Inc., Chicago, IL 60606, USA). The statistical significance level was set to *P* < 0.05 and 0.05 < *P* < 0.10 was considered demonstrating a trend for a change. Further, tests of one-sided hypotheses were deemed significant if a two-sided *P*-value was less than 0.10. The specific one-sided tests were (1) the protein contents of PDH-E1*α*  and Cyt-c are lower in MYA compared with CON; (2) basal mRNA levels of heat shock proteins HSc70 and HSP72 increase with repetitive stressful work but decrease with prolonged duration of training exercises in MYA.

## 3. Results

### 3.1. Baseline

The PDH-E1*α*  protein level was significantly lower in the MYA group than in the CON group at baseline (*P* < 0.05 in one-sided test). No other significant differences were observed between MYA and CON, and this was true for both the mRNA data and the protein data (Tables 2 and 3). Representative western blot images are presented in Figure 3. 

### 3.2. Standardized Repetitive and Stressful Work

The standardized protocol including repetitive work, stress tests, and computer work induced significant changes in the mRNA content of different genes. Significant increases were found for mRNA content of the heat shock proteins: HO-1*α*  (*P* < 0.05) and HSP72 (*P* < 0.05, one-sided), while IL-6 and HIF-1*α*  showed a trend to increase (Table 4). In contrast, PGC-1*α*, IGF-1Ea, and myostatin mRNA contents were significantly lowered (*P* < 0.05) several fold after the standardized work day (Table 4). 

### 3.3. Intervention

In the MYA group, the tenderness score was 12.5 (4–28) out of the possible maximum score of 28 at baseline and which decreased significantly in response to 10 wks intervention in SST and GFT by 11.0 (6–20) and 6.3 (−4–13), respectively. But in REF the decrease of 3.2 (−3–7) was not significant.

**Figure 1:**
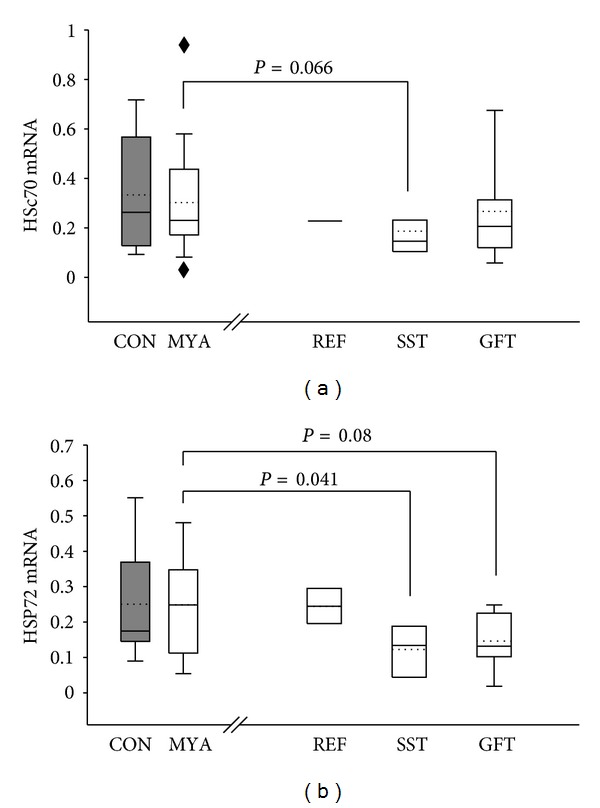
Effect of training on mRNA content. The mRNA content of HSc70 (a) and HSP72 (b) decreased in the strength training group (SST, *n* = 9) during the 10 wks intervention period (*P* < 0.05). Similarly, a decrease in HSP72 mRNA content was observed with the GFT (*n* = 10) intervention (*P* < 0.1) (B). No changes were observed for REF ((a): *n* = 2; (b): *n* = 3). The figure illustrates the 5th and 95th percentile, outliers (black dot), the group median (solid line), and group mean (dotted line).

**Figure 2:**
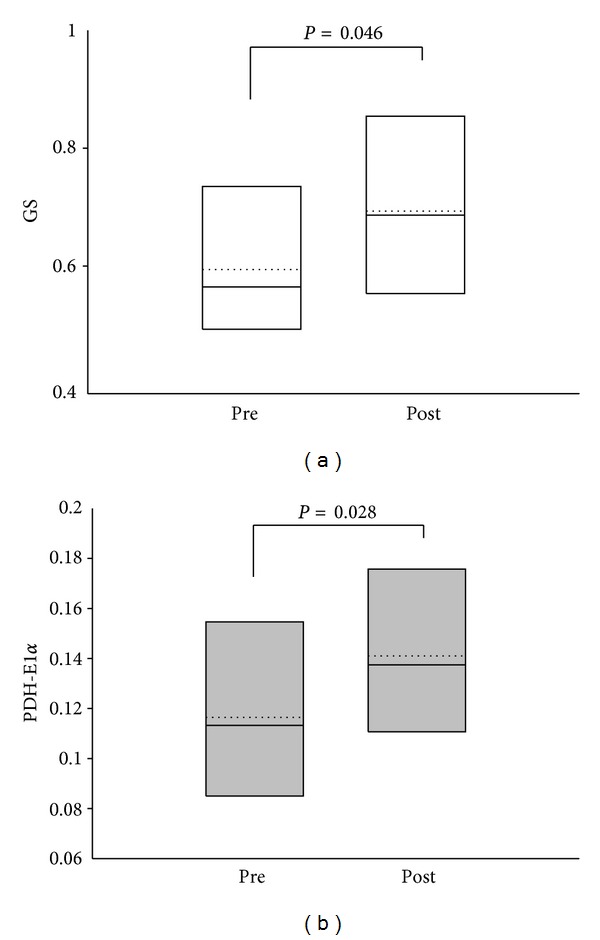
Effect of training on protein content. The protein content of GS (a) and PDH-E1*α*  (b) increased in the strength training group (SST, *n* = 6) after the 10 wks intervention period (*P* < 0.05). The figure illustrates the 5th and 95th percentile, the group median (solid line), and group mean (dotted line).

**Figure 3:**
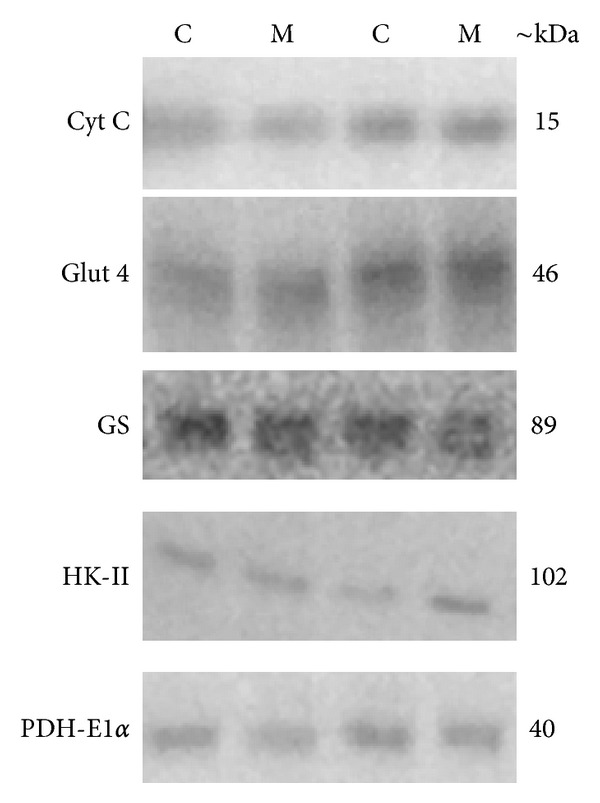
Representative western blot images at baseline for two subjects from the CON group (C) and the MYA group (M), respectively, for the proteins Cyt C, Glut 4, GS, HK-II, and PDH-E1*α*.

**Figure 4:**
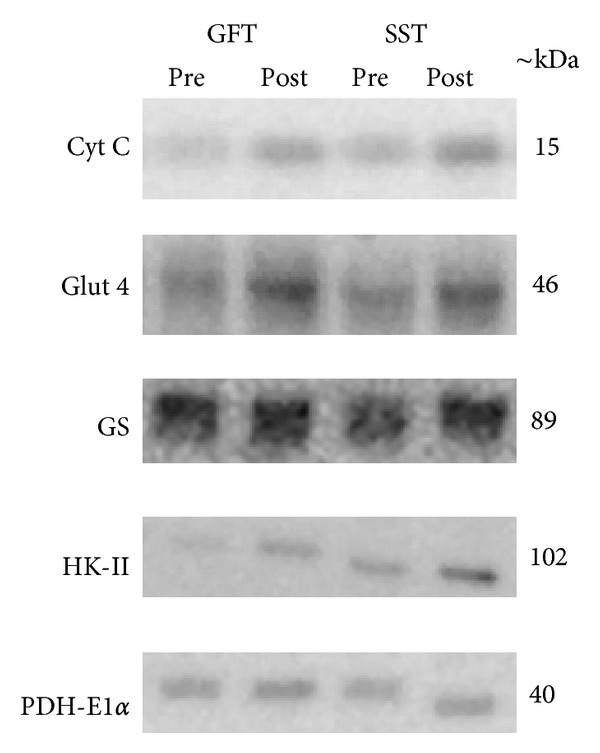
Representative western blot images at baseline (Pre) and after the intervention (Post) for subjects from the MYA group with the GFT and SST 10 wks intervention period, respectively, for the proteins Cyt C, Glut 4, GS, HK-II, and PDH-E1*α*.

Biopsy data showed the basal level of HSP72 mRNA content to decrease significantly after the SST and GFT interventions, whereas HSc70 mRNA only decreased after the SST intervention period (*P* < 0.05, one-sided) (Figures 1(a) and 1(b)). Further, GS and PDH-E1*α*  protein content increased by 22% and 21%, respectively, (*P* < 0.05) only after the SST intervention (Figures 2(a) and 2(b)). Cyt c, HKII, and GLUT4 protein content did not change in either of the intervention groups. Representative western blot images are shown in Figure 4.

## 4. Discussion

Repetitive stressful work tasks increased levels of mRNA content for heat shock proteins and decreased mRNA levels of growth regulating proteins and proteins related to oxidative metabolism. In contrast, prolonged exercise training decreased the basal level for heat shock protein. Specific strength training but not general endurance training increased the protein content of key proteins in carbohydrate storage and oxidation in myalgic skeletal muscle. Interestingly, impairment in the capacity for carbohydrate oxidation was seen at baseline in MYA compared with CON but no differences in the basal level of mRNA contents were found.

### 4.1. MYA versus CON at Baseline

A novel finding of the present study was an observed lower protein level of PDH-E1*α* in females with trapezius myalgia than healthy controls (Figure 1). PDH-E1*α*  is responsible for catalyzing the decarboxylation of pyruvate, and regulation of this PDH component of the enzyme complex is thought to be important for the mitochondrial choice of substrate at rest and during exercise [31]. Reduced capacity of the pyruvate dehydrogenase complex could facilitate movement of glycolytically derived pyruvate toward lactate output rather than oxidation. This may underlie our finding of higher interstitial muscle lactate levels and impaired muscle oxygenation in MYA compared with CON in this same experimental series during the pegboard task as reported previously [25]. This is also in accordance with a previous finding, where patients with trapezius myalgia were found to respond to repetitive low-force arm work with a larger interstitial muscle lactate increase than seen in healthy controls [32]. In concert, biopsy studies on work-related trapezius myalgia have demonstrated a reduced capillarisation per fiber cross-sectional area [33] implying an impaired oxidative capacity, while in the present subject groups such differences were not found between MYA and CON [34]. 

### 4.2. Standardized Repetitive and Stressful Work Performed by MYA

Static and highly repetitive work tasks have been identified as risk factors for work-related trapezius myalgia [35]. Patients with work-related trapezius myalgia often report that pain worsens considerably even when remarkably light physical work of neck and shoulder muscles is performed which was confirmed in the present subject group [36]. A main finding of the present study was that among MYA the experimntal work day including repetitive and stressful work tasks elicited marked changes in the mRNA abundance of a subset of metabolic and stress-related genes as will be discussed in more detail below. 

#### 4.2.1. Heat Shock Proteins

The upregulation of HSP72 mRNA content observed in the present study may confer protection against ischemia and preserve the cellular functions [37], while HSc70 mRNA content remained unchanged which is in accordance with its constitutively nature. The ~10-fold increase in HO-1 mRNA content indicates that the trapezius muscle may indeed be exposed to oxidative stress during repetitive work tasks which is in accordance with the increased interstitial lactate level reported in this subject group during the repetitive work [25]. A previous study showed that HO-1 mRNA increased with repetitive muscle contractions, and increases in HO-1 protein content in response to metabolic stress may be important in producing antioxidants [38]. In combination the observed increase in HSP72 and HO-1 mRNA levels after a work day including standardized repetitive tasks indicated that significant cellular stress was imposed to the trapezius muscle.

#### 4.2.2. Cytokines

IL-6 mRNA content was markedly upregulated after the standardized work protocol. It has been established that IL-6 is released from skeletal muscle in response to exercise [39, 40]. Even moderate exercise has major effects on muscle-derived IL-6 [41, 42]. Computer workers with neck-shoulder disorders like trapezius myalgia have shown less trapezius muscle relative rest time during work [43] and thus having a more sustained muscle activity pattern which *per se* might increase the IL-6 basal level. Furthermore, the present findings are in accordance with previous observations showing that upper extremity, low-intensity repetitive exercise results in a substantial increase in IL-6 in the interstitium of the stabilizing trapezius muscle [44]. The present finding that TNF-*α*  mRNA content did not change after the standardized work protocol is analogous to a previous study where no change in TNF-*α*  was observed after a single bout of resistance training [45]. In addition, the unchanged TNF-*α*  mRNA content may suggest that the repetitive work task did not induce an inflammatory response in chronic myalgia. This is in concert with our finding from immunohistochemical staining of the biopsies from these subjects showing similar numbers of macrophages between those with and without trapezius myalgia [27].

#### 4.2.3. Growth Regulating Proteins

In the present study, a dramatic myostatin mRNA downregulation (~90%) was observed after a work day including a standardized repetitive work protocol, which is in accordance with previous findings in response to a single exercise bout [46]. Myostatin is a member of the transforming growth factor-*β* gene family and has been shown to be a negative regulator of muscle growth [47] as it appears to inhibit satellite cell activation [48]. In contrast, IGF-I is a positive regulator of muscle growth that appears to act on several levels, including satellite cell activation [49], gene transcription, and protein translation, and seems essential in mediating the loading induced hypertrophy of skeletal muscle [50]. The expression of IGF-IEa is increased in response to several types of resistance exercise [50, 51]. However, the repetitive work tasks during the work day do not induce muscle loading corresponding to strengthening exercises [52] and which may explain the observed downregulation of IGF-IEa in the present study. The downregulations of myostatin and IGF-IEa may underlie our previous report on satellite cells showing significantly more satellite cells being associated with type I and fewer with type II muscle fibres in subjects compared to those without trapezius myalgia [27]. The type I muscle fibre-specific satellite cell response may be due to higher repetitive load in myalgia cases than in healthy controls [25, 43] and the repetitive work mainly being performed by type I muscle fibres may then have increase their satellite cell pool due to the downregulated myostatin. In contrast, the type II muscle fibre-specific satellite cell response may be due to vigorous physical activity including strengthening exercise being performed less by myalgia cases than healthy controls [27] and such activities mainly being performed by type II fibres may then have decrease their satellite cell pool due to the downregulated IGF-IEa in response to the lack of type II muscle fibre loading.

#### 4.2.4. Metabolic Regulators

PGC-1*α*  is a regulatory factor thought to coordinate the cellular response of mitochondrial proteins by regulating expression of both nuclear and mitochondrial encoded genes [53]. PGC-1*α*  mRNA content decreased after a workday including standardized repetitive work tasks. In contrast, previous studies found that PGC-1*α*  transcription and mRNA content were transiently increased by a single bout of exercise [8, 54]. The exercise-induced PGC-1*α*  upregulation is suggested to be an important mechanism modulating training-induced mitochondrial biogenesis in human skeletal muscle. Clearly, the decreased PGC-1*α*  mRNA content observed in the present study supports the notion that repetitive stressful work tasks may rather impair cellular mitochondrial adaptations. However, the present upregulation of HIF-1*α*  is consistent with other studies of increased HIF-1*α*  mRNA expression in human skeletal muscle after acute bouts of endurance exercise and may signify activation of metabolic genes that promote oxygen delivery and, most importantly, angiogenesis [55, 56]. Hypoxia has been shown to activate the transcription factor HIF-1*α*  and muscle tissue oxygenation was reduced during the repetitive work tasks as previously reported [36]. Additionally, a possible role for HIF-1*α*  to increase after a single exercise bout could be to activate the transcription of target genes, including HO-1 mRNA. The two-fold increase in HIF-1*α*  mRNA content in the present study was associated with a ~10-fold increase in HO-1 mRNA. 

### 4.3. Training Intervention for MYA

There are several novel findings in the present study. However, the main finding was that specific strength training (SST) was superior to general fitness training performed as leg-bicycling (GFT) in improving the intramuscular metabolic capacity of trapezius muscle in female with chronic neck muscle pain. Importantly, in association with the observed intramuscular metabolic adaptations marked (~70% decrease in pain score with SST) and prolonged (>10 wks after training) relieve in neck muscle pain was demonstrated in the same group of subjects as reported in a previous paper [26]. 

#### 4.3.1. mRNA Content

The potential role of HSPs in preserving cells from stress and preparing them to survive various types of environmental challenges suggests that these proteins could also play a relevant role in mediating the adaptive response of skeletal muscle to physical exercise. A novel finding of the present study was that 10 wks of specific strength training lowered the mRNA concentration of heat shock protein HSP72 and HSc70 in females with work-related trapezius myalgia. A discrepancy exists in the literature. A doubling in HSP72 protein level was reported after 5–8 wks training in previously untrained humans [57], while 12 wks high-intensity-strength training lowered the HSP72 protein level in well-trained humans [58]. Strength training is associated with changes in protein synthesis and degradation, and because heat shock proteins assist in these processes it could be expected that expression of HSP would increase initially but decline in the later phase of the training period. Mechanisms exist, which are able to attenuate the heat shock protein response [59], and the observed downregulation of HSP mRNA content in the present study could reflect an improved cellular homeostasis of trapezius muscle fibers in females with chronic neck pain.

The GFT intervention also lowered the HSP72 mRNA content. This finding supports the notion that muscle activity in one part of the body potentially affects distant muscles as well [60, 61]. Further, it has been shown that the induction of stress protein synthesis after moderate-intensity exercise occurs in both active muscle and in less active muscle, indicating that the signal which initiates the stress response may affect the whole body [62]. The existence of a generalized response of skeletal muscle to physical stress could explain the present finding that GFT—not involving exercise of the trapezius muscle—lowered the HSP72 mRNA content. Interestingly, immunohistochemical staining identifying satellites cells in biopsies from these subjects demonstrated significant increases in satellite cell content of types I and II muscle fibres following SST as well as of type II muscle fibres following GFT [63]. These findings support that strength training of the myalgic muscle has a major effect but that even training with remote muscle groups may affect the morphology of the painful trapezius muscle. This may underlie our previous finding of attenuated pain development during repetitive work tasks [36].

No changes were observed in mRNA content for the other examined genes; nevertheless, it is possible that transient responses could have occurred but were missed due to the limited number of sampling time points.

#### 4.3.2. Protein Content

GS protein expression increased with specific strength training. This is compatible with the training-induced increases in glycogen synthase protein content previously reported [64, 65]. The increase in GS protein content clearly indicates that the nonoxidative capacity for carbohydrate disposal of the trapezius muscle was improved after the SST intervention in females with chronic neck pain. The increase in PDH-E1*α*  protein content after the SST intervention indicates that not only the nonoxidative but also oxidative capacity for carbohydrate disposal was enhanced. The explanation should be found in the design of the specific strength training. As mentioned, the high-intensity program included 9 sets of 8–12 RM performed within 20 minutes which would lead to not only a nonoxidative but also an oxidative training component. The improved capacity for carbohydrate oxidation, as seen by an increase in PHD-E1*α*  protein content, would be particularly beneficial during sustained work tasks requiring prolonged muscle contractions.

### 4.4. Limitations

One limitation of the present study that must be taken into account is that the two biopsies were taken from the same incision hole in the skin before and after the standardized work day protocol. The two biopsies were taken with the needle pointing in opposite direction separating the two sampling sites as much as possible. Although this procedure has been used many times previously, we cannot rule out that the tissue may have been affected from the first biopsy taken, influencing the mRNA response. Furthermore, the small population in parallel with large variation in data may lead to a type II error. On the other hand, it may also strengthen the significant outcomes that have been identified in the present study. 

## 5. Conclusion

In conclusion, no differences were found between MYA and CON in the content of the basal level of a broad range of mRNA's. However, standardized repetitive, stressful work had great impact on the mRNA expression in MYA, indicating that daily repetitive low-intensity work tasks induce metabolic stress upon the trapezius muscle. Furthermore, a decrease in heat shock protein mRNA content was seen not only after strength training but also after general fitness training which indicates that the cellular homeostasis of the trapezius muscle was improved in females with work-related trapezius myalgia, regardless of training intervention type. However, protein content of PDH-E1*α*  was lower in MYA than CON and only specific strength training increased the capacity of the oxidative and nonoxidative carbohydrate metabolism in the trapezius muscle of MYA as seen by an increased protein level of both GS and PDH-E1*α*.

## Figures and Tables

**Table 1:** Primer and TaqMan probe sequences for real-time PCR.

Gene	Forward primer	Reverse primer	Taqman probe
GLUT4	5′ CCTGCCAGAAAGAGTCTGAAGC 3′	5′ ATCCTTCAGCTCAGCCAGCA 3′	5′ CAGAAACATCGGCCCAGCCTGTCA 3′
GS	5′ GCTCAGAGCAAGGCTCGAAT 3′	5′ CGGCCGGCGATAAAGAA 3′	5′ TTATGGGCATCTGGACTTCAACTTGGACA 3′
HIF-1*α*	5′ GCCCCAGATTCAGGATCAGA 3′	5′ TGGGACTATTAGGCTCAGGTGAAC 3′	5′ ACCTAGTCCTTCCGATGGAAGCACTAGACAA 3′
HO-1	5′ GCCAGCAACAAAGTGCAAGAT 3′	5′ AGTGTAAGGACCCATCGGAGAA 3′	5′ AGAGGGAAGCCCCCACTCAACACC 3′
Hsc70	5′ GCAGACACAGACCTTCACTACCTATT 3′	5′ GGCACGCTCGCCTTCAT 3′	5′ AACCTGAATAAGCACACCAGGCTGGTTG 3′
HSP72	5′ GCGTGATGACTGCCCTGAT 3′	5′ CGCCCTCGTACACCTGGAT 3′	5′ TCCCCACCAAGCAGACGCAGATCT 3′
IGF-IEa	5′ CAGCGCCACACCGACAT 3′	5′ TTGTTTCCTGCACTCCCTCTACT 3′	5′ AAGACCCAGAAGGAAGTACATTTGAAGAACGC 3′
IL-6	5′ TCTCAGCCCTGAGAAAGGAGACA 3′	5′ CATCTTTGGAAGGTTCAGGTTGT 3′	5′ ACATGTGTGAAAGCAGCAAAGAGGCACTG 3′
LDH-A	5′ ACAACAGGATTCTAGGTGGAGGTT 3′	5′ GAGTTGATGTTTTTCCCAGTCCAT 3′	5′ TGCATGTTGTCCTTTTTATCTGATCTGTGATTAAAGC 3′
LDH-B	5′ GCTAAAGGATGATGAGGTTGCTC 3′	5′ TCACTAGTCACAGGTCTTTTAGGTCC 3′	5′ CTCAAGAAAAGTGCAGATACCCTGTGGGAC 3′
Myostatin	5′ ACCAGGAGAAGATGGGCTGAA 3′	5′ GTCAAGACCAAAATCCCTTCTGGA 3′	5′ CCGTTTTTAGAGGTCAAGGTAACAGACACACCA 3′
PGC-1*α*	5′ CAAGCCAAACCAACAACTTTATCTCT 3′	5′ CACACTTAAGGTGCGTTCAATAGTC 3′	5′ AGTCACCAAATGACCCCAAGGGTTCC 3′
TNF*α*	5′ TCTGGCCCAGGCAGTCAGAT 3′	5′ AGCTGCCCCTCAGCTTGA 3′	5′ CAAGCCTGTAGCCCATGTTGTAGCAAACC 3′

GS: glycogen synthase; HIF-1*α*: hypoxia inducible factor-1; HO-1: heme oxygenase-1; HSP72 and HSc70: heat shock protein 70 and 72; IGF-1Ea: insulin-like growth factor-1Ea; IL-6: interleukin-6; LDH: lactate dehydrogenase; PGC-1*α*: peroxisome proliferator activated receptor gamma coactivator 1*α*; TNF-*α*: tumor necrosis factor alpha.

**Table 2:** The mRNA content for selected proteins in MYA and CON at baseline. *n* is the number of subjects for whom the specific mRNA was analyzed. Median, minimum, and maximum values are presented. No significant differences were found.

mRNA	MYA, *n *	Median	Min	Max	CON, *n *	Median	Min	Max
Glut-4	19	**0,118**	0,012	0,439	14	**0,094**	0,001	0,864
GS	18	**0,886**	0,013	4,356	9	**0,801**	0,023	2,821
HIF-1*α*	21	**0,132**	0,019	0,641	6	**0,111**	0,008	0,454
HO-1	22	**0,127**	0,008	1,608	13	**0,086**	0,023	1,615
HSc70	19	**0,230**	0,035	0,937	13	**0,263**	0,086	0,723
HSP72	17	**0,239**	0,009	0,466	12	**0,175**	0,087	0,561
IGF-1Ea	18	**0,182**	0,020	0,511	12	**0,207**	0,026	0,772
IL-6	20	**0,016**	0,005	0,072	9	**0,010**	0,002	0,164
LDH-A	15	**0,152**	0,007	0,354	1	**0,148**	0,148	0,148
LDH-B	15	**0,150**	0,025	0,291	1	**0,140**	0,140	0,140
Myostatin	21	**0,269**	0,002	1,483	13	**0,406**	0,005	1,203
PGC-1*α*	20	**0,122**	0,029	1,081	11	**0,166**	0,055	1,027
TNF-*α*	20	**0,105**	0,007	0,567	13	**0,124**	0,038	0,935

**Table 3:** Content of key metabolic proteins in for MYA and CON at baseline. *n* is the number of subjects for whom the key protein was analyzed. Median, minimum, and maximum values are presented. No significant differences were found, except for PDH-E*1α* protein being significantly lower in MYA than in CON in one-sided test (*P* < 0.05).

Proteins	MYA, *n *	Median	Min	Max	CON, *n *	Mediean	Min	Max
cyt c	19	0,142	0,069	0,212	16	0,171	0,089	0,282
Glut 4	19	0,437	0,280	0,984	16	0,393	0,280	0,774
GS	19	0,559	0,263	0,769	16	0,501	0,351	0,760
HKII	19	0,224	0,067	0,375	16	0,185	0,049	0,452
PDH-E1*α*	19	0,114	0,066	0,229	16	0,139	0,088	0,203

**Table 4:** mRNA content before and after 7 hrs standardized repetitive stressful work.

Content of mRNA	PRE	POST
HIF-1*α* (*n* = 7)^#^	0.245 (0.075–0.4859)	0.419 (0.074–3.334)
HO-1 (*n* = 8)*	0.180 (0.062–0.313)	2.069 (0.103–6.106)
HSP72 (*n* = 7)^#^	0.347 (0.239–0.614)	2.465 (0.244–3.334)
IGF-1Ea (*n* = 6)*	0.256 (0.164–0.511)	0.130 (0.108–0.208)
IL-6 (*n* = 7)^#^	0.017 (0.005–0.072)	1.244 (0.004–4.347)
Myostatin (*n* = 8)*	0.692 (0.049–1.218)	0.073 (0.011–0.610)
PGC-1*α* (*n* = 6)*	0.377 (0.092–1.082)	0.073 (0.038–0.323)

Values are presented as median (range). ^#^Denotes 0.05 < *P* < 0.10 and *denotes *P* < 0.05 in paired test. HIF-1*α*: hypoxia inducible factor-1*α*; HO-1: heme oxygenase-1; HSP72: heat shock protein 72; IGF-1Ea: insulin-like growth factor-1Ea; IL-6: interleukin-6; PGC-1*α*: peroxisome proliferator activated receptor gamma coactivator 1*α*.
